# Cytokine-Modulated Natural Killer Cells Differentially Regulate the Activity of the Hepatitis C Virus

**DOI:** 10.3390/ijms19092771

**Published:** 2018-09-14

**Authors:** Yoo Jin Cho, Hwan Hee Lee, Hyojeung Kang, Hyosun Cho

**Affiliations:** 1 Department of Pharmacy, College of Pharmacy, Duksung Women’s University, Seoul 132-714, Korea; dbwls1234567@hanmail.net (Y.J.C.); leedh010700@duksung.ac.kr (H.H.L.); 2 Duksung Innovative Drug Center, Duksung Women’s University, Seoul 132-714, Korea; 3 College of Pharmacy, Research Institute of Pharmaceutical Sciences and Institute for Microorganisms, Kyungpook National University, Daegu 702-701, Korea

**Keywords:** HCV, huh 7.5, natural killer cells

## Abstract

HCV genotype 2a strain JFH-1 replicates and produces viral particles efficiently in human hepatocellular carcinoma (huh) 7.5 cells, which provide a stable in vitro cell infection system for the hepatitis C virus (HCVcc system). Natural killer (NK) cells are large lymphoid cells that recognize and kill virus-infected cells. In this study, we investigated the interaction between NK cells and the HCVcc system. IL-10 is a typical immune regulatory cytokine that is produced mostly by NK cells and macrophages. IL-21 is one of the main cytokines that stimulate the activation of NK cells. First, we used anti-IL-10 to neutralize IL-10 in a coculture of NK cells and HCVcc. Anti-IL-10 treatment increased the maturation of NK cells by enhancing the frequency of the CD56^+dim^ population in NK-92 cells. However, with anti-IL-10 treatment of NK cells in coculture with J6/JFH-1-huh 7.5 cells, there was a significant decrease in the expression of STAT1 and STAT5 proteins in NK-92 cells and an increase in the HCV Core and NS3 proteins. In addition, rIL-21 treatment increased the frequency of the CD56^+dim^ population in NK-92 cells, Also, there was a dramatic increase in the expression of STAT1 and STAT5 proteins in rIL-21 pre-stimulated NK cells and a decrease in the expression of HCV Core protein in coculture with J6/JFH-1-huh 7.5 cells. In summary, we found that the functional activation of NK cells can be modulated by anti-IL-10 or rIL-21, which controls the expression of HCV proteins as well as HCV RNA replication.

## 1. Introduction

Hepatitis C virus (HCV) is a 9.6-kb hepatotropic RNA virus that is known to be a major cause of chronic hepatitis, liver cirrhosis, and hepatocellular carcinoma. In vivo animal models for HCV infection study are limited, but the in vitro cell culture system to study a natural HCV life cycle is well established [1,2]. In addition, a full-length HCV genome was shown to replicate and even produce infectious virus particles in a human hepatocarcinoma 7 cell line (huh 7) culture [3].

Natural killer (NK) cells are large lymphoid cells that participate in innate immune defense [4]. The major role of NK cells is killing virus-infected cells and tumor cells through abnormal or a lack of major histocompatibility antigen (MHC) I expression [5]. NK cells are identified by the expressions of CD56 and CD16 in human peripheral blood [6]. CD16 is the low-affinity Fc receptor (FcγRIIIa or FcγRIIIb) that facilitates antibody-dependent cell cytotoxicity (ADCC) [6]. The CD56^+^ populations are further divided into subsets of CD56^dim^ and CD56^bright^. The CD56^dim^ CD16^+^ subset is known to be more mature and has higher amounts of cytotoxic granules such as perforin and granzyme than the CD56^bright^ CD16^+^ subset [6]. NK cells comprise about 50% of liver-resident lymphocytes, which suggests that NK cells play crucial roles in the elimination of viral infections in the liver [4].

Resolve of HCV infection has been associated with strong HCV-specific T cell responses, whereas lack of CD4^+^ and CD8^+^ T cell responses have been observed during the chronic phase of HCV infection [7]. With regard to innate immune responses, establishment of chronic HCV infection was shown to be partly related with NK cell dysfunction, which results in the modulation of DC function or the production of immunoregulatory cytokines (TGF-β, IL-10) during HCV infection [8,9]. Although the importance of T cells and B cells against HCV infection has been well described [10], NK cell responses are relatively unclear, and there are still some arguments to be resolved [11]. In particular, a rapid and strong NK cell response early on during HCV infection is required to induce a robust T cell response against HCV that results in effective viral clearance. Meanwhile, the chronicity of HCV infection is closely connected with impairment of NK cell function [12,13].

The HCV in vitro cell culture system has been utilized to investigate the role of NK cells in HCV infection. Coculture between human primary NK cells and HCV-infected human hepatoma cells reduced the functional capacity of NK cells to degranulate as well as to target cell cytotoxicity [14].

IL-10 is a representative immune-inhibitory cytokine that has been shown to play a key role in disease progression to chronic HCV infection. Early IL-10 production in HCV-infected patients was linked with higher HCV RNA in blood, and the presence of IL-10 producing T cells was correlated with progression to chronic HCV infection [15]. Increased production of IL-10 has been suggested as a mechanism of inefficient virus-specific CD4^+^ T cell responses in chronic HCV infection [16]. Increased natural cytotoxicity receptor (NCR) expression of NK cells with IL-10 production was shown to provide a greater contribution to NK-DC crosstalk for subsequent adaptive immune responses than virus control in HCV infection [17].

Meanwhile, the important role of IL-21 in HCV infection is also well established. The frequency of HCV-specific IL-21^+^ T cells was negatively related with HCV RNA viral load in HIV/HCV co-infected patients [18]. In vitro treatment of IL-21 increased the cytolytic function of HCV-specific CD8^+^ T cells [19]. Recently, it was shown that patients with sustained virologic response (SVR) had higher pretreatment serum IL-21 levels, which suggests that the pretreatment serum IL-21 level could be a biomarker to predict SVR in chronic hepatitis C patients [20]. IFN-α pre-stimulated NK cells have been reported to kill HCV-infected hepatoma cells, which suggests that the modulation of cytokine production such as neutralizing IL-10 or adding IL-12 or IL-21 in coculture of NK cells and HCV-infected hepatoma cells could be considered as an immunotherapeutic approach against HCV infection [21]. In this study, we tried to induce the functional activation of NK cells by neutralizing IL-10 or adding exogenous IL-21 in coculture with J6/JFH1-huh 7.5 cells (HCVcc) and investigated the effect of cytokine-modulated natural killer cells on the activity of HCV. We also explored the signaling molecules that are related with NK cell activation in coculture with J6/JFH1-huh 7.5 cells.

## 2. Results

### 2.1. Effect of Anti-IL-10 on Expression of CD56, and STAT Proteins in NK-92 Cells

**Figure 1:**
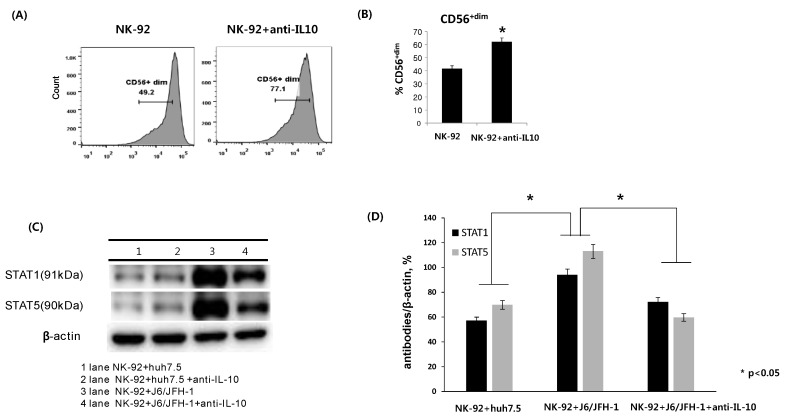
Effect of anti-IL-10 on expression of CD56, and STAT proteins in NK-92 cells. NK-92 cells were cultured in the presence or absence of anti-IL-10 (0.1 ng/mL) for 6 h and stained with fluorescent anti-CD56 antibodies. (**A**) Representative FACS plots showing CD56 expression of NK-92; (**B**) % CD56^+dim^ NK-92. Alternatively, NK-92 cells were cocultured with J6/JFH-1-huh 7.5 cells or naïve huh 7.5 cells in the presence or absence of anti-IL-10 for 6 h. Cell lysates from NK-92 cells only were assessed for the expressions of stat1 and stat5 proteins. β-actin was served as the loading control. (**C**) Expressions of STAT1 and STAT5 proteins by Western blot. (**D**) Relative band intensity of STAT1 and STAT5 compared with the loading control from at least three independent experiments.

To investigate whether neutralization of IL-10 has any effect on the surface expression of CD56 in NK-92 cells, NK-92 cells were incubated in the presence or absence of anti-IL-10 (1 ng/mL) for 6 h and stained with fluorescent anti-CD56 antibodies. The expression of CD56 on NK cells is known to be directly associated with activation-linked maturation in NK cells. There was a significant increase in the frequency of the CD56^+dim^ population of NK-92 cells (62.1%) in the presence of anti-IL-10 compared with untreated cells (41.7%) (Figure 1A,B). Subsequently, NK-92 cells were cocultured with J6/JFH-1-huh 7.5 cells or naïve huh 7.5 cells in the presence or absence of anti-IL-10 (1 ng/mL) for 6 h. Cell lysates from NK-92 cells were assessed for the expression of signal transducer and activator of transcription (STAT) proteins. The increased expressions of STAT1 and STAT5 proteins is known to be a key factor in NK cell activation. As shown in Figure 1C,D, HCV infection itself significantly increased the expressions of STAT1 and STAT5 proteins. On the other hand, neutralization of IL-10 considerably decreased the expression of STAT1 and STAT5 proteins in NK-92 cells cocultured with J6/JFH-1-huh 7.5 cells (Figure 1C,D).

### 2.2. Effect of Anti-IL-10 on Expression of HCV-Core and HCV-NS3 Proteins in Coculture with NK-92 Cells

**Figure 2:**
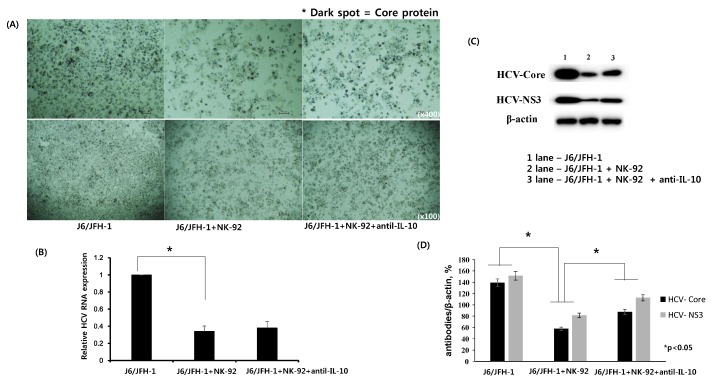
Effect of anti-IL-10 on expression of HCV Core and -NS3 proteins in coculture with NK-92 cells. J6/JFH-1-huh 7.5 cells were cocultured with NK-92 cells in the presence or absence of anti-IL-10 for 6 h. Immunohistochemical staining for HCV Core protein expression, real time qPCR for HCV RNA expression, and Western blot analysis for HCV Core and HCV NS3 proteins were performed. β-actin was served as the loading control. (**A**) Immunohistochemical expression of HCV Core protein (dark spots) in cytoplasm of huh 7.5 cells and cell nuclei were counterstained with Gill’s Hematoxylin #2 to show the absolute cell number in immunohistochemical image; (**B**) Relative HCV RNA expression level; (**C**) Expression of HCV Core and HCV NS3 proteins by Western blot; (**D**) Relative band intensity of HCV Core and HCV NS3 compared with the loading control from at least three independent experiments.

J6/JFH-1-huh 7.5 cells were cocultured with NK-92 cells in the presence or absence of anti-IL-10 for 6 h. Immunohistochemical staining for HCV Core protein expression was done with nuclei counterstain. Subsequently, real time qPCR for HCV RNA level and Western blot analysis for HCV Core and HCV NS3 proteins were performed and the results were analyzed. Fewer dark spots for cytoplasmic expression of HCV Core protein and less number of nuclei counterstain were observed in J6/JFH-1-huh 7.5 cells cocultured with NK-92 cells compared with J6/JFH-1-huh 7.5 cells in a single culture (HCV Core^+^ 59.77% in coculture; 92.49% in single culture) (Figure 2A). However, neutralization of IL-10 seems to increase the number of HCV Core positive spots as well as the number of nuclei counterstain (HCV Core^+^ 59.77% in coculture; 65.91% in anti-IL10^+^ coculture) (Figure 2A, far right). Also, HCV RNA expression showed a significant downregulation in coculture with NK-92 cells, but it was slightly upregulated in the presence of anti-IL-10 (Figure 2B). Subsequently, Western blot analysis showed that the substantial reduction of both HCV Core and NS3 proteins in coculture with NK-92 cells recovered in the presence of anti-IL-10 (Figure 2C,D), which correlates with the immunohistochemical staining for HCV Core protein as well as HCV RNA expression.

### 2.3. Effect of Anti-IL-10 on Expression of p38 and Erk in J6/JFH-1-huh 7.5 Cocultured with NK-92 Cells

**Figure 3:**
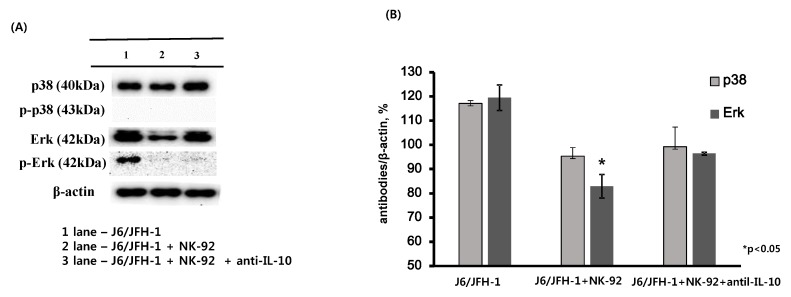
Effect of anti-IL-10 on expression of p38 and Erk in J6/JFH-1-huh 7.5 cocultured with NK-92 cells. J6/JFH-1-huh 7.5 cells were cocultured with NK-92 cells in the presence or absence of anti-IL-10 for 6 h. Western blot analysis for p38, p-p38, Erk and p-Erk proteins was performed. β-actin was served as the loading control. (**A**) Expression of p38, p-p38, Erk and p-Erk proteins by Western blot; (**B**) Relative band intensity of p38, p-p38, Erk and p-Erk proteins compared with the loading control from at least three independent experiments.

We predicted an inhibition of HCV by neutralization of IL-10 in J6/JFH-1-huh 7.5 cells cocultured with NK-92 cells because IL-10, a typical immune regulatory cytokine, was reported to link with high HCV RNA in blood from chronic HCV-infected patients. However, we observed increased expression of HCV proteins by the addition of anti-IL-10 in coculture (Figure 2A,C,D). Therefore, we further explored whether the cellular signaling molecules of J6/JFH-1-huh 7.5 cells could be affected by anti-IL-10. Western blot analysis for the expressions of p38, extracellular regulated protein kinase (Erk) proteins and the phosphorylated forms in J6/JFH-1-huh 7.5 cells cocultured with NK-92 cells (Figure 3) showed that, initially, coculture with NK-92 decreased the expressions of p38, Erk and p-Erk proteins in J6/JFH-1-huh 7.5 cells. The expression of p-p38 was not detected in any of cell cultures (Figure 3A). Interestingly, neutralization of IL-10 significantly increased the production of Erk protein, which suggests that anti-IL-10 affected not only HCV expression by modulating the activity of NK cells but also HCV-infected huh 7.5 cells (Figure 3A,B).

### 2.4. Effect of Recombinant IL-21 on Expression of CD56, STAT Proteins, and IFN-γ in NK-92 Cells

**Figure 4:**
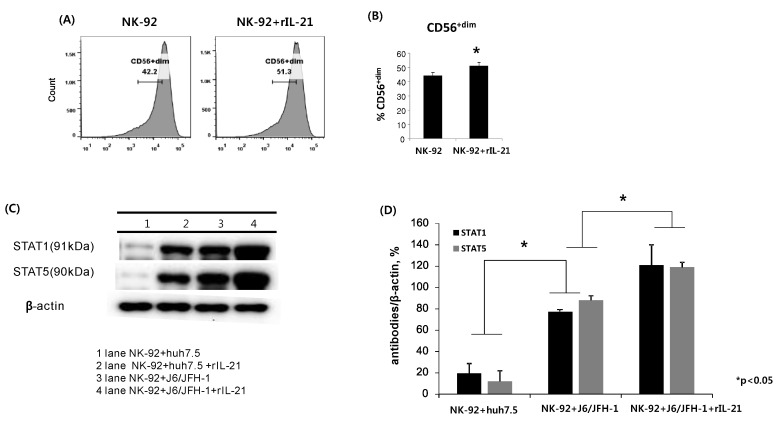

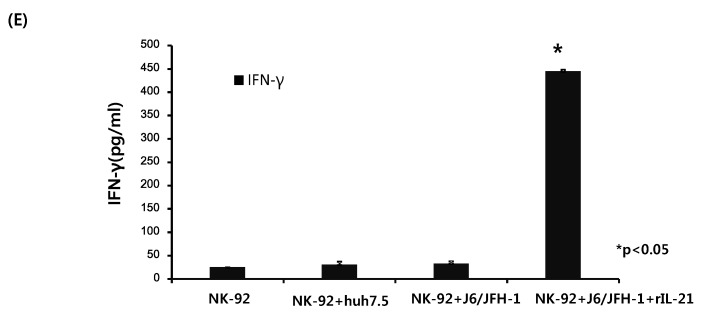
Effect of recombinant IL-21 on expression of CD56, STAT proteins, and IFN-γ in NK-92 cells. NK-92 cells were cultured in the presence or absence of recombinant IL-21 (0.2 ng/mL) for 6 h and stained with fluorescent anti-CD56 antibodies. (**A**) Representative FACS plots showing CD56 expression of NK-92; (**B**) % CD56^+dim^ NK-92. Alternatively, NK-92 cells were incubated with or without recombinant IL-21 (0.2 ng/mL) for 6 h and subsequently cocultured with J6/JFH-1-huh 7.5 cells or naïve huh 7.5 cells for 12 h. Cell lysates from NK-92 cells only were assessed for the expressions of STAT1 and STAT5 proteins. β-actin was served as the loading control. IFN-γ production (pg/mL) in culture supernatants was determined by ELISA. (**C**) Expressions of STAT1 and STAT5 proteins by Western blot; (**D**) Relative band intensity of STAT1 and STAT5 proteins compared with the loading control from at least three independent experiments; (**E**) IFN-γ production (pg/mL).

Next, we examined the effect of recombinant IL-21 on the function of NK-92 cells. NK-92 cells were cultured in the presence or absence of rIL-21 (0.2 ng/mL) for 6 h and stained with fluorescent anti-CD56 antibodies. The rIL-21 significantly increased the frequency of the CD56^dim^ population of NK-92 cells (51.1%) compared with unstimulated cells (44.2%) (Figure 4A,B). In addition, NK-92 cells were incubated with or without rIL-21 (0.2 ng/mL) pre-stimulation for 6 h and then cocultured with J6/JFH-1-huh 7.5 cells or naïve huh 7.5 cells for another 12 h. Cell lysates from NK-92 cells were assessed for the expressions of STAT1 and STAT5 proteins using Western blot. Pre-stimulation by rIL-21 dramatically increased the expressions of STAT1 and STAT5 proteins in NK-92 cells. Also, rIL-21 stimulated a significant amount of IFN-γ in NK-92 cells cocultured with J6/JFH-1-huh 7.5 cells (Figure 4E). These results indicate that rIL-21 can directly induce the activation of NK-92 cells.

### 2.5. Effect of Recombinant IL-21 on Expression of HCV-Core and -NS3 Proteins in Coculture with NK-92 Cells

We further investigated if rIL-21-pre-stimulated NK-92 cells could influence the expressions of HCV proteins and RNA replication. J6/JFH-1-huh 7.5 cells were cocultured with NK-92 cells that had or had not been pre-treated with rIL-21. Then, Immunohistochemical staining for HCV Core protein and nuclei counterstain was assessed. Also, HCV RNA level, HCV Core and HCV NS3 proteins were quantified.

**Figure 5:**
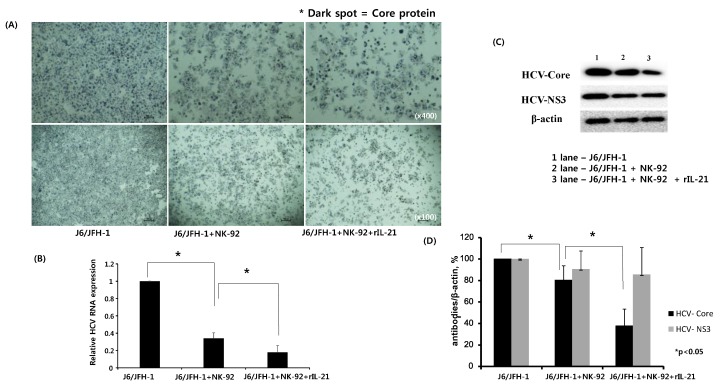
Effect of recombinant IL-21 on expression of HCV-Core and -NS3 proteins in coculture with NK-92 cells. J6/JFH-1-huh 7.5 cells were cocultured with NK-92 cells with or without IL-21 pre-stimulation. Immunohistochemical staining for HCV Core protein expression, real time qPCR for HCV RNA expression, and Western blot analysis for HCV Core and HCV NS3 proteins were performed. β-actin was served as the loading control. (**A**) Immunohistochemical expression of HCV Core protein (dark spots) in cytoplasm of huh 7.5 cells and cell nuclei were counterstained with Gill’s Hematoxylin #2 to show the absolute cell number in immunohistochemical image; (**B**) Relative HCV RNA expression level; (**C**) Expression of HCV Core and HCV NS3 proteins by Western blot; (**D**) Relative band intensity of HCV Core and HCV NS3 compared with the loading control from at least three independent experiments.

Much fewer number of dark spots (HCV Core protein) as well as and fewer number of nuclei were detected in J6/JFH-1-huh 7.5 cells cocultured with rIL-21-prestimulated NK-92 cells compared with that from coculture between J6/JFH-1-huh 7.5 cells and untreated NK-92 cells (HCV Core^+^ 93.47% in single culture; 62.07% in coculture; 51.95% in r-IL21^+^ coculture) (Figure 5A, middle and far right). HCV RNA expression level was also considerably decreased in J6/JFH-1-huh 7.5 cells cocultured with rIL-21-prestimulated NK-92 cells (Figure 5B). HCV Core protein, but not NS3 protein, was significantly reduced by rIL-21-prestimulated NK-92 cells (Figure 5C,D), which suggests that rIL-21 could control HCV infection through activating NK-92 cells.

## 3. Discussion

IL-10 is an important immunoregulatory cytokine that is produced by macrophages, Th cells, and NK cells depending on various immune environments [22]. NK cells were reported to express both IL-10 and its receptor, but the function of IL-10 on NK cell activation is not clearly understood. Tripp group suggested that IL-10 could be an antagonist for NK cell activation because of its inhibitory effect on the production of inflammatory cytokines and the activation of T cells [23]. Scott et al. showed that NK cells were significantly activated in mice treated with anti-IL-10 during peritonitis and that the activation of NK cells corresponded with decreased mice survival and the production of IFN-γ [24]. We also found that neutralizing IL-10 increased the frequency of the CD56^dim^ population of NK-92 cells, which has higher amounts of cytotoxic granules than the CD56^bright^ population (Figure 1A,B). These data indicate that IL-10 can directly inhibit the expressions of functional receptors in NK cells depending on a variety of immune milieus.

The influence of IL-10 in chronic HCV infection has been well investigated. Increased IL-10 production in chronic HCV infection positively correlated with high HCV RNA and ineffective HCV-specific T cell responses [15,16]. We examined whether neutralizing IL-10 would affect a coculture system consisting of cell culture-grown HCV (HCVcc) genotype 2a (clone JFH1) and NK-92 cells. First, the expressions of STAT1 and STAT5 proteins from NK-92 cell lysates were upregulated in coculture with J6/JFH-1-huh 7.5 cells compared with coculture with naïve huh 7.5 cells, which suggests that HCVcc induced the activation of NK-92 cells. At the same time, NK-92 seems to actively kill HCV-infected huh 7.5 cells according to a positive correlation between the decrease of dark spots for HCV core protein and reduced cell number image from nuclei counterstain (Figure 2A far left, middle and Figure 5A far left, middle). However, the increased STATs expression of NK-92 cells was significantly downregulated in the presence of anti-IL-10, which indicates that IL-10 may be required to maintain the activation of NK-92 cells through STATs expression (Figure 1C,D). It has been shown that recombinant IL-10 has a significant effect on the cytotoxicity of human NK cells and neutralizing IL-10 suppressed NK cell cytotoxicity in human NK cells. The same study also showed that recombinant IL-10 increased the expressions of STAT1 and STAT3 in NK-92 single culture, which is partly inconsistent with our data [25]. We assume that the STATs proteins of NK-92 cells in our coculture system could have complicated influences by both HCVcc as well as anti-IL10.

HCVcc (clone JFH1) has been reported to induce the production of IL-10 and inhibit DC maturation, which suggests that HCVcc could induce viral favorable immune environments by modulating host-derived IL-10 [26]. Therefore, we explored the effect of anti-IL-10 on HCVcc in coculture with NK-92 cells. We observed that coculture with NK-92 cells significantly decreased the production of HCV proteins as well as the RNA replication, which was expected because of cytotoxic effect of NK-92 cells on HCV-infected huh 7.5 cells (Figure 2B–D and Figure 5B–D). However, neutralization of IL-10 seems to restore the production of HCV Core and NS3 proteins (Figure 2A,C,D).

We previously confirmed a significant amount of IL-10 production from coculture between J6/JFH-1-huh 7.5 cells and NK-92 cells (Figure S1); therefore, we are convinced that the reduction of HCV proteins resulted from the presence of IL-10 in the coculture maintaining the activation of NK-92 cells against HCV, which supports results from the Park group [25]. However, we initially hypothesized that neutralization of IL-10 would result in HCV inhibition by activating NK cells because IL-10 was shown to be associated with high HCV RNA in blood from chronic HCV patients. Therefore, we further examined whether the signaling molecules in J6/JFH-1-huh 7.5 cells are also affected by anti-IL-10 in coculture with NK-92 cells, and we found that the downregulated Erk expression in coculture with NK-92 cells was recovered in the presence of anti-IL-10 (Figure 3A,B).

HCV viral proteins such as Core and E2 have been reported to be associated with the modulation of cell proliferation. Mitogen-activated protein kinase (MAPK) signal pathway contains the extracellular regulated protein kinase (Erk), the stress-activated protein kinase (p38), and the c-Jun N-terminal kinase (JNK), which are associated with the regulation of cellular processes including cell growth, proliferation, and apoptosis [27]. Erhardt found that both Full-length and N-truncated HCV core proteins stimulated the total protein expressions of Erk, JNK and p38 and that resulted in an increase of cell proliferation [28]. HCV E2 protein was shown to induce the phosphorylated forms of Erk in huh-7 cells [29]. Of note, the expression of phosphorylated Erk or p38 would be better indicator for the cell proliferation or activation. However, Ndjomou group reported that Erk inhibitor enhanced HCV replication by inhibiting the phosphorylation of MEK/Erk signals, which indicates that the relationship between phosphorylation status of MEK/Erk and HCV infection is still unclear [30]. Therefore, we decided to measure the protein expression of total Erk or p38 and the phosphorylated forms. Figure 3A,B show that coculture with NK-92 cells significantly decreased the expression of total Erk and p-Erk protein, which strongly suggests the cytotoxicity of NK-92 cells against J6/JFH-1-huh 7.5 cells through the Erk pathway (Figure 3A,B). Interestingly, anti-IL-10 seems to increase the production of Erk protein (Figure 3A,B), which correlates with the increased expression of HCV Core protein (Figure 2C,D). We speculate that the addition of anti-IL-10 attenuates both the cytotoxic and antiviral effect of NK-92 cells, which results in the increase of ErK protein in J6/JFH-1-huh 7.5 cells.

In addition, we tried to study the role of IL-21 in coculture between NK-92 cells and J6/JFH-1-huh 7.5 cells. Recently, IL-21 was suggested as a predictor of sustained virologic response (SVR) in chronic hepatitis C because high levels of IL-21 were clearly linked with achievement of SVR [20]. Also, HCV specific IL-21^+^ T cells were shown to be associated with HCV viral control in chronic HCV monoinfection as well as HIV/HCV coinfection [18,19]. Skak’s group reported that exogenous IL-21 increased the expression of perforin and granzyme A in human NK cells at mRNA and protein levels, even though the expressions of surface receptors on NK cells were hardly affected by IL-21 [31]. Another group, that of Wendt et al., showed that IL-21 strongly induced the proliferation of the CD56^+bright^ population in human NK cells and cytotoxicity of NK cells was increased, especially in the CD56^+dim^ population [32]. We showed that recombinant IL-21 significantly increased the frequency of CD56^+dim^, which indicates the direct augmentation of NK cytotoxicity by rIL-21 (Figure 4). This IL-21–stimulated functional activation of NK cells was maintained even in coculture with J6/JFH-1-huh 7.5 cells through the induction of STAT1 and STAT5 expressions (Figure 4C,D), but with no induction of STAT2, STAT3, or STAT4 (data not shown). It was reported that exogenous IL-21 induced the activation of STAT1, STAT3, and STAT4 but not STAT5 in human NK cells [32,33]. In fact, not only phosphorylation status of STATs but also phosphorylation site of STAT molecules differentially contributes to the regulation of cellular activation [32]. Therefore, we speculate that the presence of HCV infection differently modulated the expressions of STAT proteins, particularly STAT3, STAT4, and STAT5 of NK cells in our coculture system. The functional stimulation of NK cells by rIL-21 was significantly correlated with the production of IFN-γ, which indicates that the secretion of IFN-γ is strongly connected with the activation of STAT1 and STAT5 in our coculture experiments (Figure 4E). Serti’s group showed that primary NK cells produced a high level of IFN-γ when PBMCs were cocultured with Huh7/HCV replicon cells, which is strongly connected with the suppression of HCV replication [34]. Also, Park reported that exogenous IL-21 directly increased the cytotoxicity and IFN-γ production of ex vivo expanded NK cells in coculture with breast cancer cells [35]. Both studies indicate that anti-HCV effect of NK cells are closely associated with IFN-γ, and our results support their findings (Figure 4). Finally, we examined whether rIL-21-stimulated NK-92 cells could have a higher capacity to control the HCV infection compared with unstimulated NK-92 cells. There are still arguments about the contribution of IL-21-induced activation of NK cells in the control of viral infection. Pallikkuth et al. reported that IL-21 administration to chronic SIV-infected rhesus macaques increased the cytotoxicity of NK cells, but it had little effect on the plasma viral load [36]. Iannello’s group showed that IL-21-mediated activation of NK cells was strongly correlated with the inhibition of HIV replication [37]. Our data also showed that rIL-21-treated NK-92 cells significantly inhibited the expressions of HCV Core protein as well as HCV replication by killing HCV-infected huh 7.5 cells (Figure 5). Therefore, IL-21 could be a valuable therapeutic candidate for boosting NK cell activation in chronic HCV infection.

## 4. Materials and Methods

### 4.1. Establishment of Infectious HCV (J6/JFH1) Cell Culture System and NK-92 Cells

HCV genotype 2a-derived infectious HCV clone (pFL-J6/JFH-1) was provided from TMIN/Toray and Dr. Rice’s laboratory through a material transfer agreement (MTA 291, MTA 1464). Human hepatocarcinoma 7.5 cells (huh 7.5) were obtained from Apath, LLC through a material transfer agreement (MTA1465) and cultured in Dulbecco’s modified Eagle’s medium (DMEM; Gibco, Waltham, MA, USA) supplemented with 10% heat-inactivated fetal bovine serum (FBS; Yong-In Frontier, Seoul, Korea) and 100 U/mL penicillin and streptomycin (Gibco) at 37 °C in a humidified atmosphere with 5% CO_2_. HCV cell culture (HCVcc) was established in huh 7.5 cells by transfecting the full-length genome of J6/JFH1-derived HCV RNA, as previously described [38]. Naïve huh 7.5 cells were infected with HCV virions at 0.1 MOI (multiplicity of infection) through the experiments. Natural killer (NK-92) cells were purchased from the American Type Culture Collection (ATCC) and maintained in Minimum Essential Media alpha (alpha MEM; Gibco), added with 20% non-inactivated FBS (Yong-In Frontier) and 0.1 mM β-mercaptoethanol (Sigma, Aizu, Japan). Both cell types were incubated in the presence of 100 U/mL penicillin and streptomycin (Gibco) at 37 °C in a humidified atmosphere with 5% CO_2_.

### 4.2. Reagents

Anti-human-interleukin-10 (Anti-IL-10) and recombinant human interleukin-21 (IL-21) were purchased from BioLegend, Inc. (San Diego, CA, USA).

### 4.3. Flow Cytometry Analysis

NK-92 cells were cocultured with J6/JFH1-huh 7.5 cells in the presence of anti-IL-10 for 6 h; alternatively, NK-92 cells alone were treated with recombinant IL-21 for 6 h. After each incubation, the harvested NK-92 cells were stained with anti-CD56-APC (BD Biosciences, Franklin Lakes, NJ, USA) and analyzed by flow cytometry (Novocyte Flow Cytometer, ACEA Biosciences, San Diego, CA, USA). The positivity of CD56 was determined by comparison with the defined cutoff values obtained with unstained control cells, as previously described [39].

### 4.4. Immunohistochemistry Staining

J6/JFH1-huh 7.5 cells were plated with a density of 5 × 10^4^ cells/100 μL cells per well in 96-well plate and fixed overnight before being permeabilized in 100% methanol for 30 min at −20 °C. Cells were washed twice with PBS followed by once with 1× PBS/0.1% Tween-20 and blocked with 1% BSA and 0.2% skim milk for 30 min at room temperature. Then, 3% H_2_O_2_ was added to block endogenous peroxidase activity. Cells were stained with anti-HCV-specific Core 1b monoclonal antibody (#ab2740, Abcam, Cambridge, UK) diluted 1:2000 in 1× PBS/0.1% Tween-20 and incubated with secondary antibody (goat-a-mouse-HRP, Jackson Immuno Research) diluted 1:200 for 30 min at room temperature. DAB substrate (DAKO, K3468; diluted 1 drop/mL as per manufacturer’s instructions) was added for 5 min to detect positive dark spots. Also, nuclei counterstain was done using Gill’s Hematoxylin #2 (Polysciences, Inc., Warrington, PA, USA) to confirm the absolute cell number.

### 4.5. ELISA Assay

Cell free supernatants were harvested to measure the production of IFN-γ using a human enzyme linked immunosorbent assay kit (BD Biosciences). Absorbance was measured at 450 nm using a microplate reader (BMG Labtech, Ortenberg, Germany).

### 4.6. Western Blot Analysis

Cells were differently harvested in accordance with each experiment. The J6/JFH-1-huh 7.5 cells, naïve huh 7.5 cells, or Natural killer (NK-92) cells were individually lysed by protein extraction buffer (Intron, Gyeonggi-do, Korea). Proteins in cell lysates were measured by the Bradford assay, separated by electrophoresis, and transferred to nitrocellulose membranes, which were then incubated with 1st and 2nd antibodies. Anti-HCV core Antibody 1b (#ab2740, Abcam) and anti-HCV NS3 Antibody (#ab13830, Abcam), anti-p38 (#8690, Cell Signaling, Danvers, MA, USA), anti-p-p38 (#9215, Cell Signaling), anti-Erk (#9102, Cell Signaling), anti-p-Erk (#4370, Cell Signaling) on to huh7.5 cells or J6/JFH-1-huh 7.5 cells and anti-STAT1 (#14994S, Cell Signaling) and anti-STAT5 (#9363T, Cell Signaling) onto NK-92 cells were used for first antibodies. Blots were visualized by enhanced chemiluminescent (ECL) detection solutions (Intron).

### 4.7. Quantitative RT-PCR

RNA was extracted from J6/JFH-1-huh 7.5 cells or naïve huh 7.5 cells cocultured with NK-92 under various conditions using an RNeasy Mini kit (Qiagen, Illumina, CA, USA), and then synthesized into cDNA using M-MLV Reverse Transcriptase (BioTech, New York, NY, USA). The resulting cDNA was analyzed for the expressions of HCV genes by qRT-PCR. Gene expression levels were determined using SYBR Green reagent (Bioline, Trento, Italy) and a Step One Plus Real-time PCR system (Applied Biosystems, Foster City, CA, USA), while 18sRNA was used as an internal control. PCR primer sequences were as follows: J6/JFH1, J6/JFH1F (5′-CTTCACGCAGAAAGCGCCTA-3′)/J6/JFH1R (5′-CAAGCGCCCTATCAGGCAGT-3′).

### 4.8. Statistical Analysis

Data were processed using Microsoft Excel and the results presented as means ± SDs. Comparisons of several means were performed by one-way or two-way analysis of variance, followed by a Fisher’s exact test to identify significant differences between groups; *p*-values of less than 0.05 were considered significant.

## Supplementary Materials

The following are available online at http://www.mdpi.com/1422-0067/19/9/2771/s1.

## References

[1] Lindenbach B.D., Evans M.J., Syder A.J., Wolk B., Tellinghuisen T.L., Liu C.C., Maruyama T., Hynes R.O., Burton D.R., McKeating J.A. (2005). Complete replication of hepatitis C virus in cell culture. Science.

[2] Wakita T., Pietschmann T., Kato T., Date T., Miyamoto M., Zhao Z., Murthy K., Habermann A., Krausslich H.G., Mizokami M. (2005). Production of infectious hepatitis C virus in tissue culture from a cloned viral genome. Nat. Med..

[3] Blight K.J., McKeating J.A., Rice C.M. (2002). Highly permissive cell lines for subgenomic and genomic hepatitis C virus RNA replication. J. Virol..

[4] Doherty D.G., O’Farrelly C. (2000). Innate and adaptive lymphoid cells in the human liver. Immunol. Rev..

[5] Lee H., Kang H., Cho H. (2017). Natural killer cells and tumor metastasis. Arch. Pharm. Res.

[6] Cooper M.A., Fehniger T.A., Caligiuri M.A. (2001). The biology of human natural killer-cell subsets. Trends Immunol..

[7] Meyer-Olson D., Shoukry N.H., Brady K.W., Kim H., Olson D.P., Hartman K., Shintani A.K., Walker C.M., Kalams S.A. (2004). Limited T cell receptor diversity of HCV-specific T cell responses is associated with CTL escape. J. Exp. Med..

[8] Jinushi M., Takehara T., Tatsumi T., Kanto T., Miyagi T., Suzuki T., Kanazawa Y., Hiramatsu N., Hayashi N. (2004). Negative regulation of NK cell activities by inhibitory receptor CD94/NKG2A leads to altered NK cell-induced modulation of dendritic cell functions in chronic hepatitis C virus infection. J. Immunol..

[9] Castriconi R., Cantoni C., Della Chiesa M., Vitale M., Marcenaro E., Conte R., Biassoni R., Bottino C., Moretta L., Moretta A. (2003). Transforming growth factor β1 inhibits expression of NKp30 and NKG2D receptors: Consequences for the NK-mediated killing of dendritic cells. Proc. Natl. Acad. Sci. USA.

[10] Rehermann B. (2009). Hepatitis C virus versus innate and adaptive immune responses: A tale of coevolution and coexistence. J. Clin. Investig..

[11] Rehermann B. (2013). Pathogenesis of chronic viral hepatitis: Differential roles of T cells and NK cells. Nat. Med..

[12] Ahlenstiel G. (2013). The natural killer cell response to HCV infection. Immun. Netw..

[13] Tatsumi T., Takehara T. (2016). Impact of natural killer cells on chronic hepatitis C and hepatocellular carcinoma. Hepatol. Res..

[14] Yoon J.C., Lim J.B., Park J.H., Lee J.M. (2011). Cell-to-cell contact with hepatitis C virus-infected cells reduces functional capacity of natural killer cells. J. Virol..

[15] Flynn J.K., Dore G.J., Hellard M., Yeung B., Rawlinson W.D., White P.A., Kaldor J.M., Lloyd A.R., Ffrench R.A., Group A.S. (2011). Early IL-10 predominant responses are associated with progression to chronic hepatitis C virus infection in injecting drug users. J. Viral. Hepat..

[16] Rigopoulou E.I., Stefanidis I., Liaskos C., Zervou E.K., Rizos C., Mina P., Zachou K., Syrganis C., Patsidis E., Kyriakopoulos G. (2005). HCV-RNA qualitative assay based on transcription mediated amplification improves the detection of hepatitis C virus infection in patients on hemodialysis: Results from five hemodialysis units in central Greece. J. Clin. Virol..

[17] De Maria A., Fogli M., Mazza S., Basso M., Picciotto A., Costa P., Congia S., Mingari M.C., Moretta L. (2007). Increased natural cytotoxicity receptor expression and relevant IL-10 production in NK cells from chronically infected viremic HCV patients. Eur. J. Immunol..

[18] MacParland S.A., Fadel S.M., Mihajlovic V., Fawaz A., Kim C., Rahman A.K., Liu J., Kaul R., Kovacs C., Grebely J. (2016). HCV specific IL-21 producing T cells but not IL-17A producing T cells are associated with HCV viral control in HIV/HCV coinfection. PLoS ONE.

[19] Feng G., Zhang J.Y., Zeng Q.L., Jin L., Fu J., Yang B., Sun Y., Jiang T., Xu X., Zhang Z. (2013). HCV-specific interleukin-21^+^CD4^+^ T cells responses associated with viral control through the modulation of HCV-specific CD8+ T cells function in chronic hepatitis C patients. Mol. Cells.

[20] Ragab H.M., El-Maksoud N.A., Amin M.A., Halim M.H., Abdulla N.A., Kamel A., Moussa S.M. (2017). IL-21 as a Predictor of Sustained Virologic Response in Patients with Chronic Hepatitis C Virus Infection. Appl. Biochem. Biotechnol..

[21] Stegmann K.A., Bjorkstrom N.K., Ciesek S., Lunemann S., Jaroszewicz J., Wiegand J., Malinski P., Dustin L.B., Rice C.M., Manns M.P. (2012). Interferon α-stimulated natural killer cells from patients with acute hepatitis C virus (HCV) infection recognize HCV-infected and uninfected hepatoma cells via DNAX accessory molecule-1. J. Infect. Dis..

[22] Moore K.W., O’Garra A., de Waal Malefyt R., Vieira P., Mosmann T.R. (1993). Interleukin-10. Annu. Rev. Immunol..

[23] Tripp C.S., Wolf S.F., Unanue E.R. (1993). Interleukin 12 and tumor necrosis factor α are costimulators of interferon γ production by natural killer cells in severe combined immunodeficiency mice with listeriosis, and interleukin 10 is a physiologic antagonist. Proc. Natl. Acad. Sci. USA.

[24] Scott M.J., Hoth J.J., Turina M., Woods D.R., Cheadle W.G. (2006). Interleukin-10 suppresses natural killer cell but not natural killer T cell activation during bacterial infection. Cytokine.

[25] Park J.Y., Lee S.H., Yoon S.R., Park Y.J., Jung H., Kim T.D., Choi I. (2011). IL-15-induced IL-10 increases the cytolytic activity of human natural killer cells. Mol. Cells.

[26] Saito K., Ait-Goughoulte M., Truscott S.M., Meyer K., Blazevic A., Abate G., Ray R.B., Hoft D.F., Ray R. (2008). Hepatitis C virus inhibits cell surface expression of HLA-DR, prevents dendritic cell maturation, and induces interleukin-10 production. J. Virol..

[27] Dinsmore C.J., Soriano P. (2018). MAPK and PI3K signaling: At the crossroads of neural crest development. Dev. Biol..

[28] Erhardt A., Hassan M., Heintges T., Haussinger D. (2002). Hepatitis C virus core protein induces cell proliferation and activates ERK, JNK, and p38 MAP kinases together with the MAP kinase phosphatase MKP-1 in a HepG2 Tet-Off cell line. Virology.

[29] Zhao L.J., Zhang X.L., Zhao P., Cao J., Cao M.M., Zhu S.Y., Liu H.Q., Qi Z.T. (2006). Up-regulation of ERK and p38 MAPK signaling pathways by hepatitis C virus E2 envelope protein in human T lymphoma cell line. J. Leukoc. Biol..

[30] Ndjomou J., Park I.W., Liu Y., Mayo L.D., He J.J. (2009). Up-regulation of hepatitis C virus replication and production by inhibition of MEK/ERK signaling. PLoS ONE.

[31] Skak K., Frederiksen K.S., Lundsgaard D. (2008). Interleukin-21 activates human natural killer cells and modulates their surface receptor expression. Immunology.

[32] Wendt K., Wilk E., Buyny S., Schmidt R.E., Jacobs R. (2007). Interleukin-21 differentially affects human natural killer cell subsets. Immunology.

[33] Strengell M., Sareneva T., Foster D., Julkunen I., Matikainen S. (2002). IL-21 up-regulates the expression of genes associated with innate immunity and Th1 response. J. Immunol..

[34] Serti E., Werner J.M., Chattergoon M., Cox A.L., Lohmann V., Rehermann B. (2014). Monocytes activate natural killer cells via inflammasome-induced interleukin 18 in response to hepatitis C virus replication. Gastroenterology.

[35] Park Y.K., Shin D.J., Cho D., Kim S.K., Lee J.J., Shin M.G., Ryang D.W., Lee J.S., Park M.H., Yoon J.H. (2012). Interleukin-21 increases direct cytotoxicity and IFN-γ production of ex vivo expanded NK cells towards breast cancer cells. Anticancer Res..

[36] Pallikkuth S., Rogers K., Villinger F., Dosterii M., Vaccari M., Franchini G., Pahwa R., Pahwa S. (2011). Interleukin-21 administration to rhesus macaques chronically infected with simian immunodeficiency virus increases cytotoxic effector molecules in T cells and NK cells and enhances B cell function without increasing immune activation or viral replication. Vaccine.

[37] Iannello A., Boulassel M.R., Samarani S., Tremblay C., Toma E., Routy J.P., Ahmad A. (2010). IL-21 enhances NK cell functions and survival in healthy and HIV-infected patients with minimal stimulation of viral replication. J. Leukoc. Biol..

[38] Lee S., Lee H.H., Kim J., Jung J., Moon A., Jeong C.S., Kang H., Cho H. (2015). Anti-tumor effect of Cordyceps militaris in HCV-infected human hepatocarcinoma 7.5 cells. J. Microbiol..

[39] Lee H.H., Kang H., Cho H. (2017). Recovery of NK(CD56^+^CD3^−^) cells after one year of tenofovir therapy for chronic hepatitis B infection. J. Microbiol. Biotechnol..

