# Supplementary material for: Cytokine-Modulated Natural Killer Cells Differentially Regulate the Activity of the Hepatitis C Virus
Source: Int J Mol Sci. 2018 Sep 14;19(9):2771. doi: 10.3390/ijms19092771 (PMC6163477; doi:10.3390/ijms19092771)
Supplement: Supplementary file 1 [file ijms-19-02771-s001.pdf]

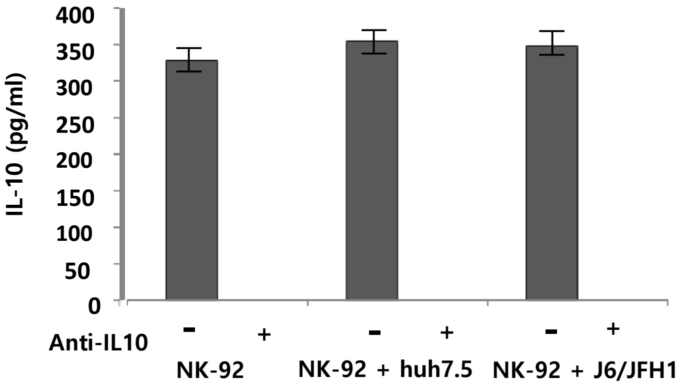

Supplementary Figure 1. Neutralization of IL-10 by anti-IL-10 treatment. NK-92 cells alone or coculture with huh7.5 cells (or J6/JFH1) were incubated in the presence or absence of anti-IL-10 (0.1 ng/ml) for 6 h and supernatants from each culture was harvested and the amount of IL-10 was measured using ELISA.
